# Thermophilic bacterial communities inhabiting the microbial mats of “indifferent” and chalybeate (iron‐rich) thermal springs: Diversity and biotechnological analysis

**DOI:** 10.1002/mbo3.560

**Published:** 2017-12-15

**Authors:** Ramganesh Selvarajan, Timothy Sibanda, Memory Tekere

**Affiliations:** ^1^ Department of Environmental Sciences College of Agriculture and Environmental Sciences UNISA Science Campus Florida South Africa

**Keywords:** 16S rRNA gene, biotechnology, microbial diversity, microbial mats, thermal springs, thermophiles

## Abstract

Microbial mats are occasionally reported in thermal springs and information on such mats is very scarce. In this study, microbial mats were collected from two hot springs (Brandvlei (BV) and Calitzdorp (CA)), South Africa and subjected to scanning electron microscopy (SEM) and targeted 16S rRNA gene amplicon analysis using Next Generation Sequencing (NGS). Spring water temperature was 55°C for Brandvlei and 58°C for Calitzdorp while the pH of both springs was slightly acidic, with an almost identical pH range (6.2–6.3). NGS analysis resulted in a total of 4943 reads, 517 and 736 OTUs for BV and CA at, respectively, a combined total of 14 different phyla in both samples, 88 genera in CA compared to 45 in BV and 37.64% unclassified sequences in CA compared to 27.32% recorded in BV. Dominant bacterial genera in CA microbial mat were *Proteobacteria* (29.19%), *Bacteroidetes* (9.41%), *Firmicutes* (9.01%), *Cyanobacteria* (6.89%), *Actinobacteria* (2.65%), *Deinococcus‐Thermus* (2.57%), and Planctomycetes (1.94%) while the BV microbial mat was dominated by *Bacteroidetes* (47.3%), *Deinococcus‐Thermus* (12.35%), *Proteobacteria* (7.98%), and *Planctomycetes* (2.97%). Scanning electron microscopy results showed the presence of microbial filaments possibly resembling *cyanobacteria*, coccids, rod‐shaped bacteria and diatoms in both microbial mats. Dominant genera that were detected in this study have been linked to different biotechnological applications including hydrocarbon degradation, glycerol fermentation, anoxic‐fermentation, dehalogenation, and biomining processes. Overall, the results of this study exhibited thermophilic bacterial community structures with high diversity in microbial mats, which have a potential for biotechnological exploitation.

## 1 INTRODUCTION

Terrestrial hot springs support diverse groups of thermophilic prokaryotes whose presence and survival is largely determined by temperature and other abiotic factors including pH, redox potential, and hydrogen sulfide concentrations (Lau, Aitchison, & Pointing, 2009). Microbial mats have been found to develop in a wide range of thermal habitats including hot springs, fumaroles, eruption vents, and on steaming ground (Pagaling et al., 2012). The majority of these microbial mats are organic rich, vertically laminated, cosmopolitan communities which are tolerable to extreme conditions (Borsodi et al., 2012). While microbial mats support a diverse range of microbial communities (Lau et al., 2009), temperature is a major factor in determining the dominating microbial mat members, especially the presence or absence of oxygenic and anoxygenic phototrophs (Coman, Drugǎ, Hegedus, Sicora, & Dragoş, 2013). For example, an investigation into the composition of microbial mats from two hot springs with a temperature range of 60–65°C and a pH range of 6.5–8.5 in China revealed the presence of diverse bacterial populations belonging to *Cyanobacteria, Chloroflexi, Chlorobia, Nitrospirae, Deinococcus‐Thermus, Proteobacteria, Firmicutes, Bacteroidetes,* and *Actinobacteria* (Pagaling et al., 2012). In contrast, Anda et al. (2015) investigated the population characteristics of thermophilic prokaryotic communities inhabiting the biofilms of a thermal karst system containing relatively high amounts of sulfide (215.3 mg/L), a temperature of 74°C and a slightly acidic pH (pH 6.2) and found it to be dominated only by sulfur oxidizing bacteria belonging to the genus *Sulfurihydrogenibium*. Research focusing on microbial diversity of mats has been on the increase in different parts of the world (Anda et al., 2015; Coman et al., 2013; Lau et al., 2009) because the key findings show that these microbial mats have a wide range of microbial communities with potential for ecological importance and biotechnological applications in aquaculture, bioremediation, agriculture, and energy production (Mostafa & El‐Gendy, 2013).

Environmental microbial populations interact with each other forming complex, mixed communities (Guerrero, Piqueras, & Berlanga, 2002), even in extreme conditions. Over the past few decades, the understanding of microbial diversity in different environments has greatly improved owing to advances in molecular phylogenic studies (Hugenholtz et al., 1998). Identification of 16S ribosomal RNA sequences from different environments has revealed that bacterial diversity has, to date, been classified into 52 different phyla (Garza & Dutilh, 2015). This type of analysis has also revealed that more than 99% of bacterial sequences belong to unknown bacteria (Temperton & Giovannoni, 2012) that have not yet been cultured in the laboratory. Although massive efforts are taken to cultivate different classes of bacteria from complex (extreme) habitats, a promising complementary approach is needed to access the genetic information of these habitats through culture independent techniques (Ramganesh, Maredza, & Tekere, 2014). Next‐generation sequencing (NGS) provides new opportunities in environmental science and technology and, coupled with advanced bioinformatics tools, has enabled rapid progress in microbial ecology and discovery of novel genes (Temperton & Giovannoni, 2012). Furthermore, it has opened up opportunities for the discovery of new “organisms” and the exploration of the distribution and roles of organisms in extreme environments (Ramganesh et al., 2014). However, some recent findings suggest that a number of environments are yet to be explored, pointing to a possibility that environmentally important microbial diversities are yet to be studied (Abed, Klempova, Gajdos, & Certik, 2015).

South Africa has numerous thermal springs that represent topographically driven meteoric water migrating along major fault zones (Magnabosco et al., 2014). There are approximately 90 identified thermal springs within South Africa (Olivier, Venter, & Jonker, 2011). Some thermal springs and other ecological habitats like freshwater systems, intertidal and subtidal marine zones, lagoons, and saline ponds and lakes are usually inhabited by microbial mats, which are essentially sedimentary biofilms (Rasuk et al., 2016). Brandvlei (64°C) is one of the hottest thermal springs in South Africa (Olivier & Jonker, 2013). This spring has been classified as an “*indifferent”* thermal water system, a term used to describe mineral water containing only a small quantity of saline matter (Boekstein, 2012). Calitzdorp, another South African thermal spring of importance to this study is classified as a “chalybeatic” thermal system, a term which denotes mineral spring waters containing high salts of iron (Olivier et al., 2011). Relatively few studies on the geochemical and bacterial diversity analysis of thermal springs have been done in South Africa, most of them in Limpopo Province (Magnabosco et al., 2014). According to Lau et al. (2009), there are major gaps in our knowledge of the overall diversity and abundance of thermophilic taxa within microbial mats. To the best of our knowledge, there are no documented studies on the thermophilic bacterial diversity adapted to microbial mats of thermal springs in the Western Cape Province of South Africa. Therefore, the aim of this study was to understand the microbial diversity and compare the structure of thermophilic bacterial communities of the microbial mats collected from two different hot springs in the Western Cape Province of South Africa using culture independent tools like next generation sequencing (NGS), scanning electron microscopy (SEM) as well as analysis of physicochemical characteristics.

## 2 MATERIALS AND METHODS

### 2.1 Spring location and sampling

**Figure 1:**
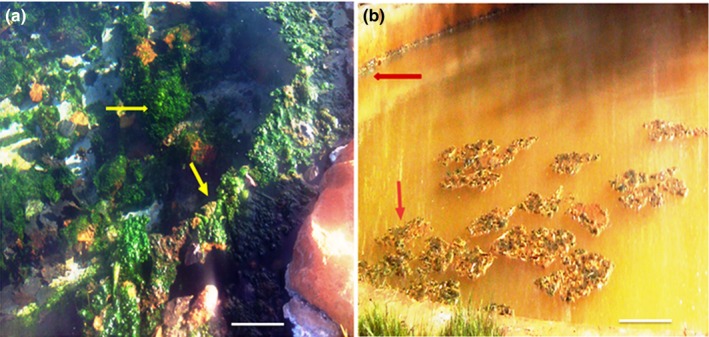
The studied microbial mats from thermal springs, Western Cape Province (South Africa) (a) Brandvlei (indifferent water system)—greenish mat—marked with arrow (b) Calitzdorp (Chalybeate water system) greenish‐brown mat—marked with an arrow (Scale bar‐10 cm)

There are 11 thermal springs in the Western Cape Province, seven of which have been developed into resorts focused on health and wellness, with appropriate facilities including Calitzdorp spa (Boekstein, 2012). Brandvlei Hot Spring is located at 33°43′56.3″S 19°24′49.4″E while Calitzdorp Spa is situated at 33°39′37.9″S 21°46′25.4″E 39. Two (2) liter water samples from each site were collected into a clean sterile glass container for the analysis of major ions (Silicon, Calcium, Potassium, Magnesium, Sodium, Fluoride, Chloride, and Bromide) while water for trace metal analysis (listed in Table 1) was collected into acid washed glass containers. For the Brandvlei mat, the water, which is up to 0.5 m deep around the corner of the springs, has a temperature of 52°C. Around the edge of the pool are numerous rocky outcrops that are covered with microbial mats that form bush‐like structures (Figure 1a). The mat sample about 10 cm below the water surface was excised from its point of attachment from the stones with the help of a sterile scalpel. However, for Calitzdorp, a man‐made concrete wall was built around the spring pool. The water in this spring looks brown colored due to its high iron content. The microbial mats were found over the surface of the water and also layered on the wall (Figure 1b). The surface temperature was about 58°C. Samples layered on the wall surface (about 10 cm below the surface) were rich in green layer and were scrapped using a sterile scalpel. Both mat samples were collected in sterile glass containers for microscopic and molecular analysis. Both water and biofilm samples were stored at 4°C and transported to the laboratory for analysis within 12 hr of collection. Mat samples were collected from two different hot springs in the Western Cape Province located on the south‐western tip of South Africa in June 2015. No access and sampling restrictions were placed on the study sites and the field studies also did not involve endangered or protected species and no animals and human research subjects were involved in this study.

**Table 1:** Physicochemical and biological characteristics of the water samples collected from Brandvlei and Calitzdorp springs (Western Cape Province, South Africa)

Parameters	Units	Brandvlei Hot spring (BV)	Calitzdorp Spa (CA)
**Physicochemical**			
Temperature	°C	55 (57)	58 (44)
pH	–	6.2 (5.9)	6.28 (6.8)
Dissolved oxygen	mg/L	1.85 (n.d)	3.01 (n.d)
Conductivity	μS/cm	110 (n.d)	237 (n.d)
Salinity	Ppt	ND	0.11 (n.d)
TDS	mg/L	55 (47)	109 (115)
ORP	mV	153 (n.d)	−63.5 (n.d)
**Nutrients**			
Soluble Iron	mg/L	<0.1 (n.d)	3 (n.d)
Total Iron	mg/L	0.2	3.04 (0.8)
Nitrate	mg/L	0.16 (0.7)	0.005
Nitrite	mg/L	<0.001 (n.d)	<0.001 (n.d)
Total Sulfur	mg/L	<0.2 (n.d)	1.96 (n.d)
**Majority ions**			
Silicon	mg/L	4.9 (19.4)	4.77 (41.2)
Calcium	mg/L	4.25 (2.4)	10.8 (9.1)
Potassium	mg/L	1.69 (2.3)	5.89 (9.2)
Magnesium	mg/L	2.16 (2.6)	4.66 (4.7)
Sodium	mg/L	9.7 (9.0)	20.6 (17.5)
Fluoride	mg/L	0.1 (0.2)	0.28
Chloride	mg/L	15.8 (14.5)	30.3 (40.6)
Bromide	mg/L	0.08 (n.d)	0.09 (n.d)
**Trace Elements**			
Silver	mg/L	Trace	Trace
Aluminum	mg/L	Trace	Trace
Arsenic	mg/L	Trace	Trace(t)
Boron	mg/L	0.22	Trace
Barium	mg/L	Trace (t)	0.23
Beryllium	mg/L	Trace (n.d)	Trace (n.d)
Bismuth	mg/L	Trace (n.d)	Trace (n.d)
Molybdenum	mg/L	Trace	Trace
Nickel	mg/L	Trace (t)	0.22(t)
Lead	mg/L	Trace	Trace
Selenium	mg/L	Trace	Trace
Strontium	mg/L	0.22 (t)	Trace (0.1)
Lead	mg/L	Trace	Trace
Tellurium	mg/L	Trace (n.d)	0.22 (n.d)
Vanadium	mg/L	Trace	Trace
Zinc	mg/L	Trace	Trace
**Other parameters**			
DIC	ppm	2.15 (n.d)	8.47 (n.d)
DOC	ppm	0.84 (n.d)	0.4 (n.d)

Measurable quantities of trace elements lower than <0.2 mg/L considered as “Trace”.

ND, Not detected; n.d, Not determined; (t)‐trace quantities lower than <0.1 mg/L.

Values in the bracket were measured in 2012 obtained from Boekstein (2012).

TDS‐total dissolved solids, ORP‐oxygen redox potential, DIC‐dissolved inorganic carbon, DOC‐dissolved organic carbon.

### 2.2 Determination of physical and chemical parameters of the water

Physicochemical parameters such as temperature, pH, conductivity, salinity, total dissolved solids (TDS), oxidation redox potential (ORP), and dissolved oxygen (DO) were measured and recorded *on site* during sampling using a Multi‐parameter Meter (Hanna Instruments PTY LTD, Johannesburg, RSA). In addition, total iron, total sulfur, and soluble iron were also recorded (in situ) using hand held probes and kits (CHEMetrics, Inc. U.S.A). Major ions were quantified following the methodology of American Public Health Association (APHA, 2001) while trace metals were determined using inductively coupled plasma‐mass spectrometry (ICP‐MS).

### 2.3 Electron microscopy

The microbial mats were analyzed using scanning electron microscopy (SEM). The mats were fixed overnight using Karnovsky's fixative (8% *v/v* formaldehyde and 16% *v/v* glutaraldehyde and 0.2M PBS) at 4°C. The fixed samples were washed twice with saline phosphate buffer and with ethanol (30% *v/v*) for 5 min following the descriptions of Rasuk et al. (2016), shock frozen in liquid nitrogen and freeze dried overnight. After lyophilization, the samples were mounted on metal stubs (aluminum holder) and coated with 10 nm gold using high resolution sputter coater. The samples were then examined using a JOEL (JSM‐IT 300) scanning electron microscope at an accelerating voltage of 20 kV followed by the SEM. Elemental composition of the collected mats was determined by energy‐dispersive X‐ray (EDX) analysis operated at 20 mÅ and 20 kV at the Nano Science research unit (University of South Africa, Science Campus, South Africa). The samples were measured from 2° to 40° 2θ, with a scan speed of 0.04°/s.

### 2.4 DNA Extraction and sequencing

Microbial mat samples (about 0.5 g each) were subjected to total DNA extraction using the Quick *g*‐DNA Extraction Kit^™^ (Zymo Research Corporation, USA) according to the manufacturer's protocol. Total DNA was eluted and quantified using a NanoDrop spectrophotometer (Nanodrop 2000, Thermo Scientific, Japan). Polymerase chain reaction (PCR) was performed on the extracted DNA samples using the universal bacterial primers 27F and 518R targeting the variable region V1‐V3 of the 16S ribosomal DNA. PCR reactions contained 25 μl of one *Taq* 2X Master Mix, 22 μl of Nuclease‐free water, 1.5 μl of both forward and reverse primers at a concentration of 0.2 μmol/L and 2 μl of extracted DNA (50–100 ng μl^−1^) to make up a volume of 50 μl. The thermal profile consisted of an initial denaturation step at 95°C for 10 min, followed by 32 cycles of denaturation at 95°C for 30 s, annealing at 55°C for 30 s and extension at 72°C for 1 min, and a final extension at 72°C for 10 min, followed by cooling to 4°C. PCR amplicons were purified using a DNA Clean & Concentrator Kit (Zymo Research Corporation, USA). Following the purification step, the pooled PCR products were sequenced on the GS‐FLX‐Titanium series 454/Roche by Inqaba Biotechnology (Pretoria, South Africa).

### 2.5 Sequence data analysis

The raw sequence data‐set was initially analyzed by removing low‐quality score reads with a cutoff of 25 for Phred quality score. Next, the chimeric sequences were identified and removed using UCHIME following the descriptions of Edgar, Haas, Clemente, Quince, and Knight (2011). The resultant nonchimeric sequences were analyzed using the ribosomal database project (RDP) pipeline classifier tool with a confidence threshold of 80% following the method of Wang, Garrity, Tiedje, and Cole (2007). Taxonomic classification was performed using the composition‐based RDP classifier (v 2.2) with a bootstrap cutoff of 50%, which allowed ~95% accuracy on identification at the genus level to assign taxonomy by the Naïve Bayesian classification method. Genetic distance was determined and sequences were clustered into operational taxonomic units (OTUs) using a 0.03 dissimilarity cut‐off level. The nonparametric diversity indices including Shannon–Weaver index and the Chao1 richness estimator were calculated at the genetic distance of 0.03 to measure the diversity of bacterial species among the data‐sets. The percentage of relative abundance of individual taxa within each community was estimated by comparing the number of sequences assigned to a specific taxon against the number of total sequences obtained for that sample. The sequence datasets were deposited in the National Center for Biotechnology Information (NCBI), under accession number SAMN06250058/1677. The bio‐project can also be accessed in NCBI under Bio project ID PRJNA362842.

## 3 RESULTS AND DISCUSSION

### 3.1 Physico‐chemical characteristics

Water temperature was 55°C in Brandvlei (BV) and 58°C in Calitzdorp (CA), marking an increase in water temperature in CA thermal spring from 2012 to 2015 (see Table 1). One possible explanation is that as the water passes through fractures and fissures in subterranean rock formations, there is pressure build‐up that results in water heating up (Erfurt‐Cooper & Cooper, 2009). It therefore follows that should there be an increase in the volume of water passing through these fissures with no corresponding increase in fissure openings, there is likely to be pressure build up that will translate into higher water temperature. These thermal conditions could be favoring the proliferation of thermophilic microbial communities as also suggested by Anda et al. (2015) who indicated that microbial community composition is highly correlated with physicochemical parameters, including temperature. The pH in both springs was slightly acidic, with a range of 6.2–6.3. pH data values from 2012 to 2015 did not indicate any significant variation during this period in both springs. The concentration of TDS levels in BV thermal spring was 55 mg/L, falling into the category of simple thermal water or “*indifferent*” thermal system (Figure 1a), which are characterized by TDS levels less than 100 mg/L. In comparison, the CA spring recorded TDS levels of 109 mg/L, and 3.04 mg/L of iron, which makes the water brownish in color (Figure 1b), earning the spring the name ferruginous (iron rich) or “*chalybeatic”* thermal system.

Comparatively, the overall mineral content of BV was relatively lower than that of CA with the exception of silicon. Usually, thermal springs are mineralized to a greater or lesser degree depending on the physiognomies of the geological formations linked with the groundwater circulation (Olivier et al., 2011). The measured redox potential values also differed between the two thermal springs, with BV showing a positive value of about 150 mV and CA a value of −63.5 mV (reductive environment). Redox plays an important role in the solubility of nutrients, especially metal ions, and microbial activity. Aerobic microbial communities have been observed to be very active at positive redox values, whereas strict anaerobes are active at negative redox values (Husson, 2013). However, the facultative microbial communities will adjust their metabolic activity with or without oxygen‐bearing inorganic compounds such as nitrates and sulfates (Husson, 2013). The levels of most trace elements in both springs were below detection limit. However, BV contained detectable amounts of boron and strontium whereas CA had detectable levels of barium, nickel and tellurium, as shown in Table 1. In both springs, elemental composition and concentration were within prescribed limits as determined by the Department of Water Affairs and Forestry, South Africa for springs used for health purposes.

### 3.2 SEM–EDS analysis

**Figure 2:**
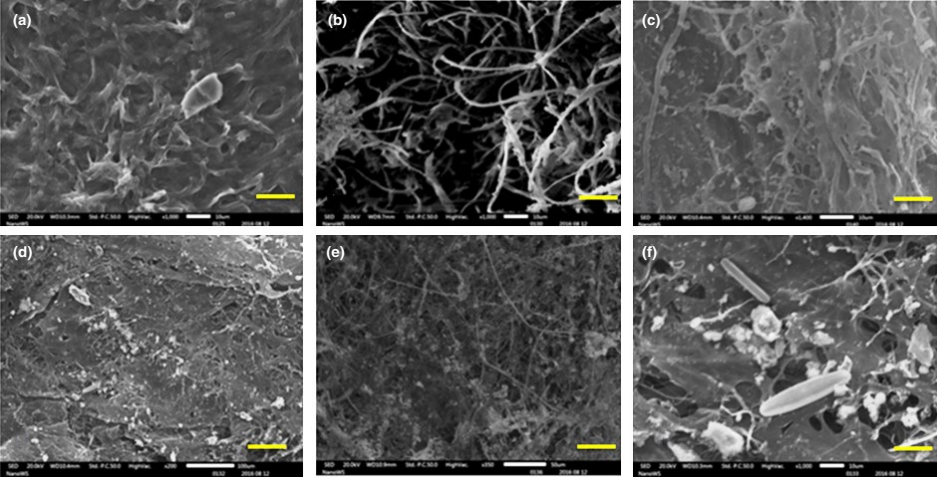
SEM photomicrographs of collected Microbial Mat; a‐c Brandvlei microbial mat (a) surface of the microbial mat covered by EPS (b) microbial filaments (c) close‐up of filamentous and cocci‐shaped cells; d‐f Calitzdorp microbial Mat (d) surface layer (e) intertwined microbial filaments in matrix of EPS (f) close‐up of filamentous and rod‐shaped cells and diatoms attached on EPS. Scale bar a, b, c, f‐10 μm; e‐50 μm, and d‐100 μm

Microbial mats are organo‐sedimentary structures that can hold diverse microbial groups including archaea, bacteria (including cyanobacteria), fungi, diatoms, microalgae, and sometimes protozoans (Borsodi et al., 2012). In this study, scanning electron microscopy analysis of collected microbial mats revealed the surface morphology of microbial filaments resembling cyanobacterial filaments (Figure 2b), coccids (Figure 2c–e), and rod‐shaped bacteria and diatoms (Figure 2f) along with extracellular polymeric substances (EPS) and mineral deposits. Since both springs were high temperature (>50°C) systems, EPS are known to play a vital role in cell protection against damage by the extreme temperature and ultra‐violet (UV) radiation (De Philippis, Margheri, Materassi, & Vincenzini, 1998). In addition to cell protection, EPS have been reported to act as carbon and energy reserves as well as being functional elements in the adhesion and immobilization of cells within microbial mats (Coman et al., 2013).

In general, microbial mats maintain optimal chemical and physiological conditions including solute concentration and redox potential which allows the cells to improve mineralization processes (Husson, 2013). Microbial mat samples from BV and CA were also compared for mineral deposition by SEM‐Energy dispersive X‐ray spectroscopy (EDS). Results of EDS analysis revealed large proportions of carbon deposited in BV mats compared to CA mats (S. Figure S1). Calitzdorp mats were observed to have higher amounts of ferrous iron and silica deposition than BV mats. According to Pierson and Parenteau (2000), the presence of high levels of ferrous iron and silica creates a sinter deposit of distinctive color and composition in the emerging waters while the microbes present in the mat contribute actively and/or passively to the mineral precipitations of iron. Calitzdorp also recorded higher quantities of calcium. Though calcification is a common phenomenon in geochemical process, microbial mats play an important role which is largely influenced and controlled by the microbes present in a mat (Shelf, Ascaso, Wierzchos, Ferna, & Quesada, 2004).

### 3.3 Sequence analysis

In this study, thermophilic bacterial community structures and species richness in the metagenome from microbial mats of the two thermal springs resulted in a total of 4,943 reads generated from both mat samples (Table 2). The sequences were searched for identity with rRNA gene sequences, and 3,062 sequences showed ribosomal origin. In total, 399 chimeric sequences were removed and 2,663 high‐quality nonchimeric rRNA reads were obtained and considered for further analysis. The mean read length was 272 and 255 bp for Brandvlei and Calitzdorp mat samples, respectively, and these were successfully classified at the domain level.

**Table 2:** Diversity indices from microbial mat collected from two different thermal springs

Sample ID	BV	CA
Total number of Sequences	2214	2729
High‐quality nonchimeric reads	1150	1513
Mean Sequences Length (bp)	272 ± 48	255 ± 67
No of OTUs at 0.03	517	736
Chao‐1 at 0.03	2169.3	3956.9
Shannon index at 0.03	5.7	6.4

### 3.4 Community species richness and diversity indices

**Figure 3:**
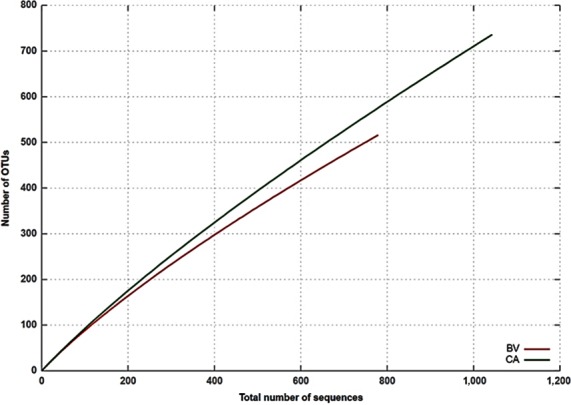
Rarefaction curves using 0.03 dissimilarity cut‐off levels; BV‐Brandvlei microbial mat and CA‐Calitzdorp microbial mat

The ribosomal database project (RDP) pipeline was used to determine the bacterial community composition at the genetic distance level of 0.03 nucleotide cutoff. A total of 517 and 736 OTUs were obtained for BV and CA at 3%, respectively. In order to determine the microbial complexity between and within the collected microbial mats, the Shannon–weaver and Chao1 index were determined at three cutoffs (Table 2). Shannon diversity index is the most widely used index for diversity comparison between habitats (Clarke & Warwick, 2001). In this study, the Shannon diversity index showed nearly similar microbial diversity in both microbial mats with 5.7 for BV and 6.4 for CA at 3% cutoff, respectively. In the same way Chao1 index was calculated to estimate the species richness based on rare OTUs within the collected mats at 3% nucleotide cutoff. However, the estimated species richness index indicated higher bacterial richness at 3% cutoff for CA when compared with BV microbial mat. The higher number of OTUs obtained for the metagenome of Calitzdorp microbial mat could be due to the increase in complexity of the thermophilic communities with increases in levels of some physicochemical parameters like temperature, DO, TDS, and total Fe in CA as compared to BV. The rarefaction curves (Figure 3) revealed that the diversity of microbes could be even more complex than witnessed, and similar findings were reported in other microbial mats including hypersaline mat (Abed et al., 2015), geothermal mat (Coman et al., 2013) and hydrothermal vent microbial mat (Headd & Engel, 2014).

### 3.5 Thermophilic bacterial communities

In order to compare the thermophilic bacterial communities from the two different microbial mats, the RDP classifier tool was used to classify the reads into phylum phylogenic fingerprints. Excluding the unclassified bacterial sequences, 10 bacterial phyla were recorded in CA while 11 phyla were recorded in BV mat sample, giving a combined total of 14 different phyla in both samples. From these, 18 bacterial classes were recorded in CA compared to 15 for BV, 39 orders in CA compared to 24 in BV, 58 families in CA compared to 29 in BV, and finally, 88 genera in CA compared to 45 in BV. CA recorded the highest proportion of unclassified sequences at 37.64% compared to 27.32% recorded in BV. The occurrence of unclassified sequences can be difficult to explain, more so when a targeted 16S rRNA gene analysis was carried out. However, unclassified sequences are not an uncommon occurrence as they have previously been reported in other studies like that of Badhai, Ghosh, and Das (2015) who reported 43.8% unclassified sequences, Delgado‐Serrano et al. (2014) who reported a maximum of 30.7% unassigned sequences in their study and Bhatia et al. (2015) who reported 31% unclassified sequences. To a large extent, occurrence of unclassified sequences have been attributed to lack of reference sequences or use of a strict Lowest Common Ancestor (LCA)‐taxonomic assignment algorithm (Badhai et al., 2015). The rank abundance curve signified taxonomic richness and evenness representing the abundance of hitherto unclassified sequences (Figure S2) which supports the finding that relatively, there is a high abundance of uncultivable microbes in any environment (Aslam, Yasir, Khaliq, Matsui, & Chung, 2010). The occurrence of unclassified sequences in this study suggests that these thermal springs are a potential reservoir of diverse microbial life which is yet uncharacterized.

**Figure 4:**
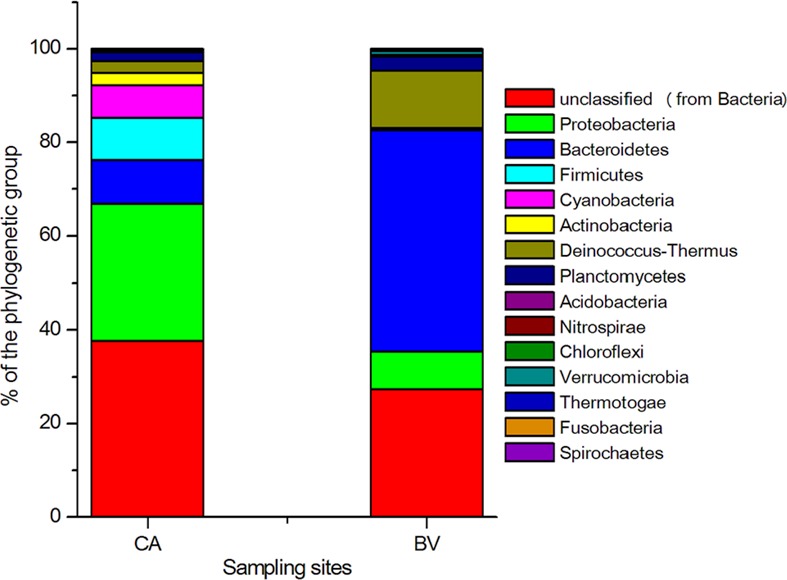
Taxonomic distribution of different bacterial phylogenetic groups in Brandvlei (BV) and Calitzdorp (CA) microbial mat samples. The percentages of the phylogenetically classified sequences are plotted on the yaxis. 16S rRNA gene sequences were classified based on the RDP 16S rRNA gene database (E value, 0.01; minimum alignment length, 50 bp)

In terms of their abundance in CA: BV; *Proteobacteria* were 29.19%: 7.98%, *Bacteroidetes* (9.41%: 47.3%), *Firmicutes* (9.01%), *Cyanobacteria* (6.89%: 0.39%), *Actinobacteria* (0.03%: 2.65%), *Deinococcus‐Thermus* (2.57%: 12.35%), and *Planctomycetes* (1.94%: 2.97%) (Figure 4). In other studies with almost similar findings with this study, Badhai et al. (2015) assessed the taxonomic and functional characteristics of four geothermal springs in Odisha, India, and showed that the metagenome sequence data of all four springs with a temperature range of 40–58°C (and pH 7.2–7.4) were dominated by bacteria over archaea, with the most abundant phyla being *Chloroflexi* and *Proteobacteria*. In yet another study to taxonomically characterize the planktonic microbial communities of Neotropical Andes hot springs (28–68°C) in Colombia, Delgado‐Serrano et al. (2014) reported overall dominance by the bacterial phyla *Proteobacteria* over others such as *Aquificae*,* Chloroflexi*,* Cyanobacteria*,* Firmicutes*,* Nitrospirae,* and *Thermotogae*. Representatives of *Thermotogae, Fusobacteria, Streptophyta, Spirochaetes,* and *Verrucomicrobia* were only found in BV microbial mat while *Firmicutes, Chloroflexi,* and *Acidobacteria* were found only in the CA microbial mat.

### 3.6 Proteobacteria

*Proteobacteria* was the dominant phyla in CA mat samples. Within this phylum, the most abundant class was *Gammaproteobacteria* (21.64%) followed by *Betaproteobacteria* (6.68%), other classes which occurred in low percentages included *Alphaproteobacteria* (0.38%), *Deltaproteobacteria* (0.15%), and *Epsilonproteobacteria* (0.34%) (Table S1). Some previous studies have demonstrated that *Epsilonproteobacteria* is dominant in microbial habitats associated with sulfidic springs (45°C) and hydrothermal vent (> 100°C), and plays a role in carbon and sulfur cycling (Headd & Engel, 2014). Compared to CA, the BV mat sample was comprised of *Gammaproteobacteria* (5.9%), *Betaproteobacteria* (1.35%), *Alphaproteobacteria* (0.55%), and *Deltaproteobacteria* (0.16%) while *Epsilonproteobacteria* was not recorded (Table S1). Elsewhere, the abundance of *Proteobacteria* has been reported in microbial mats from different regions (Abed et al., 2015; Coman et al., 2013) and many extreme environments such as hydrothermal vents (170°C) and deep mines. At genus level, the unclassified bacterial sequences were the most abundant in CA mat sample at 37.63% followed by *Pseudomonas* spp. (21.35%). This was not surprising given the findings Borsodi et al. (2012) who also found the existence of mats dominated by noncyanobacterial organisms and even fungi in marine sediments and thermal karst systems. Moreover, *Pseudomonas* spp. is well‐known to form biofilms due to their metabolic versatility in extreme conditions (Bonilla‐Rosso et al., 2012). While constituting only 21.35% and 5.9% of bacterial sequences in the CA and BV mat, respectively*, Pseudomonas* has also been reported in thermal springs in India where, importantly, the presence of *Pseudomonas stutzeri* and *Acidovorax* spp. was linked to the possible presence of genes associated with hydrocarbon degradation pathways in the Anhoni hot springs (43.5–55°C) (Saxena et al., 2017). Presumptively, this implies that thermal springs are potential reservoirs of bacterial species that can be used in the bioremediation of polluted environments.

Other dominant genera belonging to *Proteobacteria* included *Acidovorax* (1.93%), *Janthinobacterium* (1.47%), and *Trichococcus* spp. (1.04%), all detected in the CA mat sample. Previously, Pikuta et al. (2006) isolated members of the genus *Trichococcus* only from low temperature environments and ours becomes the first report of *Trichococcus* in thermal springs. Members of this genus have been reported to be able to ferment glycerol without yeast extract and produce propanediol, making it attractive for biotechnological applications (van Gelder, Aydin, Alves, & Stams, 2012). *Janthinobacterium* spp. have commonly been isolated from soil and aquatic samples, and are well‐known for their antifungal properties (Haack et al., 2016). The genus *Acidovorax* was reclassified from the genus *Pseudomonas* and comprises nearly 15 species. It has been reported to associate itself with bloom forming cyanobacteria (Chun et al., 2017) in aquatic environments. Members of this genus contain temperature resistant proteins that allow them to grow under thermophilic conditions, and were recently detected as one of the most abundant species in a thermal spring (47°C) in Coamo, Rico (Valle & Rios‐Velazquez, 2016).

### 3.7 Bacteroidetes

**Figure 5:**
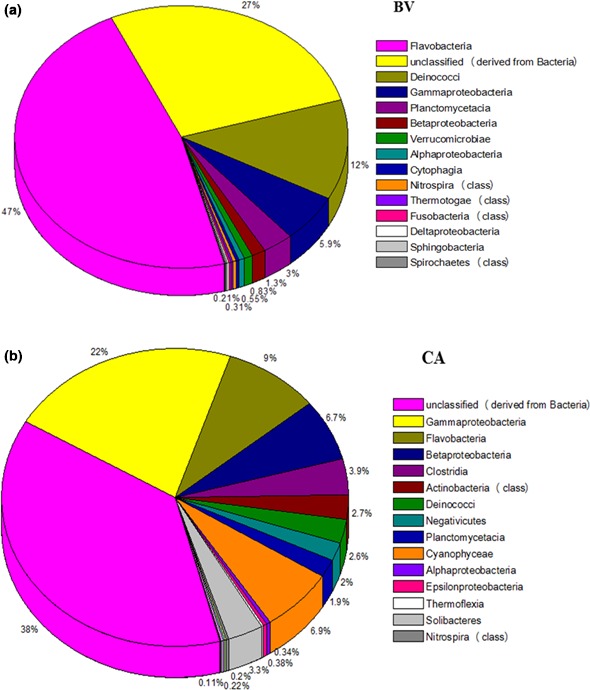
The class‐level diversity and distribution of the bacterial OTUs identified in the 16S rRNA (a) Brandvlei mat sample (b) Calitzdorp mat sample

In the phylum *Bacteroidetes,* three classes including *Flavobacteria* (BV, 47.3%; CA, 8.95%), *Sphingobacteria* (BV, 0.14%; CA, 0.11%), and *Cytophagia* (BV, 0.31%; CA, 0.13%) were recorded in both mat samples while *Bacteroidia* (CA, 0.22%) was recorded only in CA mat sample (Figure 5; Table S1). Similar results have been reported for microbial mats obtained from hydrothermal vents in the Mid Atlantic Ridge (Crepeau et al., 2011). *Deinococci* from the phylum *Deinococcus‐Thermus* was more abundant in BV (12.35%) than CA (2.57%).

*Flavobacterium* constituted 8.95% and 28.4% of bacterial sequences derived from CA and BV mat samples, respectively. While the presence of *Flavobacterium* in thermal environments has been described elsewhere (Saiki, Kimura, & Arima, 1972), some species are pathogenic and their presence in recreational springs may be a cause of public health concern. For instance, *Flavobacterium meningosepticum* is a known human pathogen that causes meningitis (Baker, Gaffar, Cowan, & Suharto, 2001). Members of the genus *Myroides* (16.02%) were reported only in the BV microbial mat. These are widely distributed in the environment due to their ability to auto aggregate and co‐aggregate to form biofilms (Elantamilan, Lyngdoh, Choudhury, Khyriem, & Rajbongshi, 2015). Some members of this genus have been reported as etiologic agents for invasive infection, especially in the immunocompromised (Beharrysingh, 2017).

### 3.8 Firmicutes

The phylum *Firmicutes* was represented by members of the genera *Clostridium* (3.91%), *Sporomusa* (2.12%), and *Desulfitobacterium* (1.58%), all of which were present in CA mat sample. The occurrence of *Clostridium* spp. in thermal habitats has previously been described (Stainthorpe & Williams, 1988). In other settings, Panda, Bisht, De Mandal, and Kumar (2016) has described the presence of *Clostridium* in Jakrem (Meghalaya), an alkaline Indian hot spring with water temperature of 46°C and pH 9 where it was present as one of the major genera. While its occurrence in swimming pools and spas has been associated with pathogenism (Martinez, 2004), other findings indicate that some species have been used for the simultaneous saccharification and fermentation of lignocellulosic biomasses (Stainthorpe & Williams, 1988) and, when such *Clostridium* was isolated from moderately thermal springs (50–60°C), it was found to be useful for production of ethanol and butyrate (Orlygsson, Sigurbjornsdottir, & Bakken, 2010). Similarly, the members of *Sporomusa* from sediments of Great Basin hot springs (40–78°C) in the USA were also reported as potential candidates of an anaerobic ethanol‐producing cellulolytic bacteria (Zhao et al., 2014). Based on these reports, it is evident that anaerobic oxidizing thermophiles are of potential biotechnological interest for the anaerobic fermentation of synthesis gas (“syngas”). *Desulfitobacterium* strains were reported to be dominantly present in acidic hot springs (pH 2.7) of El Coquito (Jimenez et al., 2012). Most of these strains can dehalogenate organic compounds by mechanisms of reductive dehalogenation, and can be excellent candidates for the development of anaerobic bioremediation processes (Villemur, Lanthier, Beaudet, & Lépine, 2006). Furthermore, characterization of anaerobic redox bacteria in geothermal systems, therefore, could provide fundamental information about the anaerobic oxidation and reduction system available for these biotechnological applications.

### 3.9 Deinococcus–Thermus

Members of the genus *Meiothermus* have been isolated from natural hot springs and artificial thermal environments in many parts of the world including Russia, France, Portugal, China, Taiwan, Iceland and the Azores (Sikorski et al., 2010) in addition to South Africa (this study) where it made up 12.35% and 2.45% of bacterial sequences in the BV and CA mat samples, respectively. In another study, Spanevello and Patel (2004) successfully characterized some *Meiothermus* species from brown, green and red microbial mats of an Australian subsurface aquifer with temperatures ranging from 52°C to 66°C. Li et al. (2014) reported the strain *Meiothermus taiwanensis* WR‐220 to produce a novel enzyme galactokinase which can tolerate up to 75°C, attracting substantial research attention due to its potential application in the enzymatic preparation of unique sugar phosphates.

### 3.10 Cyanobacteria

*Cyanobacteria* are distributed in all types of ecological niches from cold environments to hot springs (Ramganesh et al., 2014) where they constitute important components responsible for the formation of microbial mat tissue with interlaced filaments (Krienitz et al., 2003). In this study, the dominant cyanobacterial genera detected in CA mats included *Arthospira* (3.41%), *Anabaena* (2.85%)*, Nostoc* (0.27%), and *Synechococcus* (0.12%), whereas the only cyanobacterial genera encountered in BV mat sample were *Synechococcus* (0.22%) and *Nostoc* (0.17%). Since dissolved oxygen (DO) is one of the critical parameters in relation to cyanobacterial abundance (Rasuk et al., 2016), higher DO concentrations in CA (3.01 mg/L) compared to BV (1.85 mg/L) could have contributed to higher cyanobacterial densities in CA compared to BV. Most cyanobacterial genera have a worldwide distribution and are known to produce cyanotoxins that could pose public health risks, especially if present in springs that are used as spas for therapeutic purposes. However, the cyanobacterium *Anabaena* has been shown to survive in habitats where fluctuations between saline and freshwater environments occur frequently by accumulating sucrose internally when exposed to saltwater and secreting it into the external milieu when exposed to freshwater (Pfeffer & Brown, 2016). These sucrose osmolytes are a potential raw material for bioethanol production. Besides this study, the presence of *Anabaena* spp. has previously been reported in hot water springs elsewhere (Alcamán et al., 2017) and also in soda pits (Khabubu & Gumbo, 2014). Another cyanobacteria identified in this study was *Arthrospira* spp. commonly known as *Spirulina*. Like *Anabaena*,* Arthrospira* is also known for producing cyanotoxins, the casualties of which have mostly been wildlife (Krienitz et al., 2003). However, it has a range of economic uses including its use as a food supplement (owing to its high protein and vitamin content), production of pharmaceuticals and nutraceuticals, bioenergy and extraction of bio‐oil (Mostafa & El‐Gendy, 2013).

### 3.11 Other important thermophiles

Other retrieved bacterial sequences showed that the CA (chalybeatic) thermal spring harbors populations of sulfur and iron reducing/oxidizing *Proteobacteria* belonging to the genera *Acidiphilium, Acidithiobacills, Desulfomicrobium, Desulfosporosinus, Desulfotomaculum,* and *Desufuromonas* (Table S2). Occurrence of these members was likely influenced by the presence of iron and sulfur in CA spring water (Table 1) and also supported by SEM‐EDS analysis confirming that iron metabolizing bacteria may have potential significance in these chalybeatic springs. While Johnson and Hallberg (2003) classified those members of *Proteobacteria* such as *Acidiphilium* and *Acidithiobacills* as mesophilic bacteria. The findings here suggest that these bacteria can tolerate high temperature and can be classified as moderate thermophiles. BV mat sample also harbored members of iron‐oxidizing bacteria *Leptothrix*, sulfur‐oxidizing and lithotrophic bacteria belonging to the genera *Thiobacillus* and *Halothiobacillus* which were also reported in thermal springs (43°C and pH 7.6) of Bath, England (Wood & Kelly, 1988). In contrast, the CA mat contained minor fractions of sequences related to nonsulfur diazotrophic bacterium *Oscillochloris (Chloroflexi)* which is capable of carbon dioxide fixation via the reductive pentose phosphate cycle (Kuznetsov et al., 2011). Due to their role in oxidation–reduction (redox) processes, these members are actively engaged in carbon and sulfur cycles and can be excellent candidates for bio‐mining and bioremediation processes (Johnson & Hallberg, 2003).

## 4 CONCLUSION

This study presented bacterial diversity analysis of microbial mat samples collected from the hot springs of Brandvlei (BV) and Calitzdorp (CA) using next generation sequencing technology (pyrosequencing). The findings show that the most dominant phyla inhabiting the CA and BV microbial mats were *Proteobacteria* and *Bacteroidetes,* respectively. The major genera found in CA (with sequence coverage of between 1.04 and 37.64%) were *Pseudomonas, Flavobacterium, Clostridium, Arthospira, Anabaena, Meiothermus, Sporomusa, Acidovorax, Desulfitobacterium, Planctomyces, Janthinobacterium,* and *Trichococcus* while those found in BV included *Flavobacterium*,* Myroides*,* Meiothermus*,* Pseudomonas,* and *Planctomyces* in the range 1.45% to 28.4%. The occurrence of unclassified sequences (CA: 37.64% and BV: 27.72%) suggests that these thermal springs are a potential reservoir of diverse microbial life which is yet uncharacterized. To the best of our knowledge, this work presents the first report on the preliminary investigation of microbial diversity of collected mats from the environmental sites under study here. Moreover, this study will contribute the understanding of thermophile diversity and ecology of terrestrial geothermal ecosystems for biotechnological interest.

## CONFLICTS OF INTEREST

The authors declare no conflict of interest.
