# Supplementary material for: Thermophilic bacterial communities inhabiting the microbial mats of “indifferent” and chalybeate (iron‐rich) thermal springs: Diversity and biotechnological analysis
Source: Microbiologyopen. 2017 Dec 15;7(2):e00560. doi: 10.1002/mbo3.560 (PMC5911995; doi:10.1002/mbo3.560)
Supplement: Supplementary file 1 [file MBO3-7-na-s001.docx]

**Supplementary material**

**Title: Thermophilic bacterial communities inhabiting the microbial mats of ‘indifferent’ and chalybeate (iron-rich) thermal springs: diversity and biotechnological analysis**

**Running Head: Thermophiles from microbial mats for biotechnology**

1. **Ramganesh Selvarajan***, Department of Environmental Sciences, College of Agriculture and Environmental Sciences, UNISA Science Campus, Republic of South Africa. Email address: [ramganesh.presidency@gmail.com](mailto:ramganesh.presidency@gmail.com)
2. **Timothy Sibanda**, Department of Environmental Sciences, College of Agriculture and Environmental Sciences, UNISA Science Campus, Republic of South Africa. Email address: [timsibanda@gmail.com](mailto:timsibanda@gmail.com)
3. **Memory Tekere**, Department of Environmental Sciences, College of Agriculture and Environmental Sciences, UNISA Science Campus, Republic of South Africa. Email address: [tekerm@unisa.ac.za](mailto:tekerm@unisa.ac.za)

*Corresponding author email: ramganesh.presidency@gmail.com

**Supplementary Captions**

S. Fig 1: Energy dispersive X-ray spectroscopy (EDS) shows the presence and relative quantity of different elements (a) Brandvlei mat (b) Calitzdorp mat.

S Fig 2: Rank abundance plots: (a) Brandvlei microbial mat and (b) Calitzdorp microbial mat. The plots show the taxonomic abundances ordered from the most abundant to least abundant. The y-axis plots the % of abundances of annotations in each taxonomic group.

Table S1: Relative abundance (%) of major classes in different phylogenetic groups.

Table S2: Taxonomic designation of the OTUs (%) obtained from two different microbial mats.

**S. Fig 1**


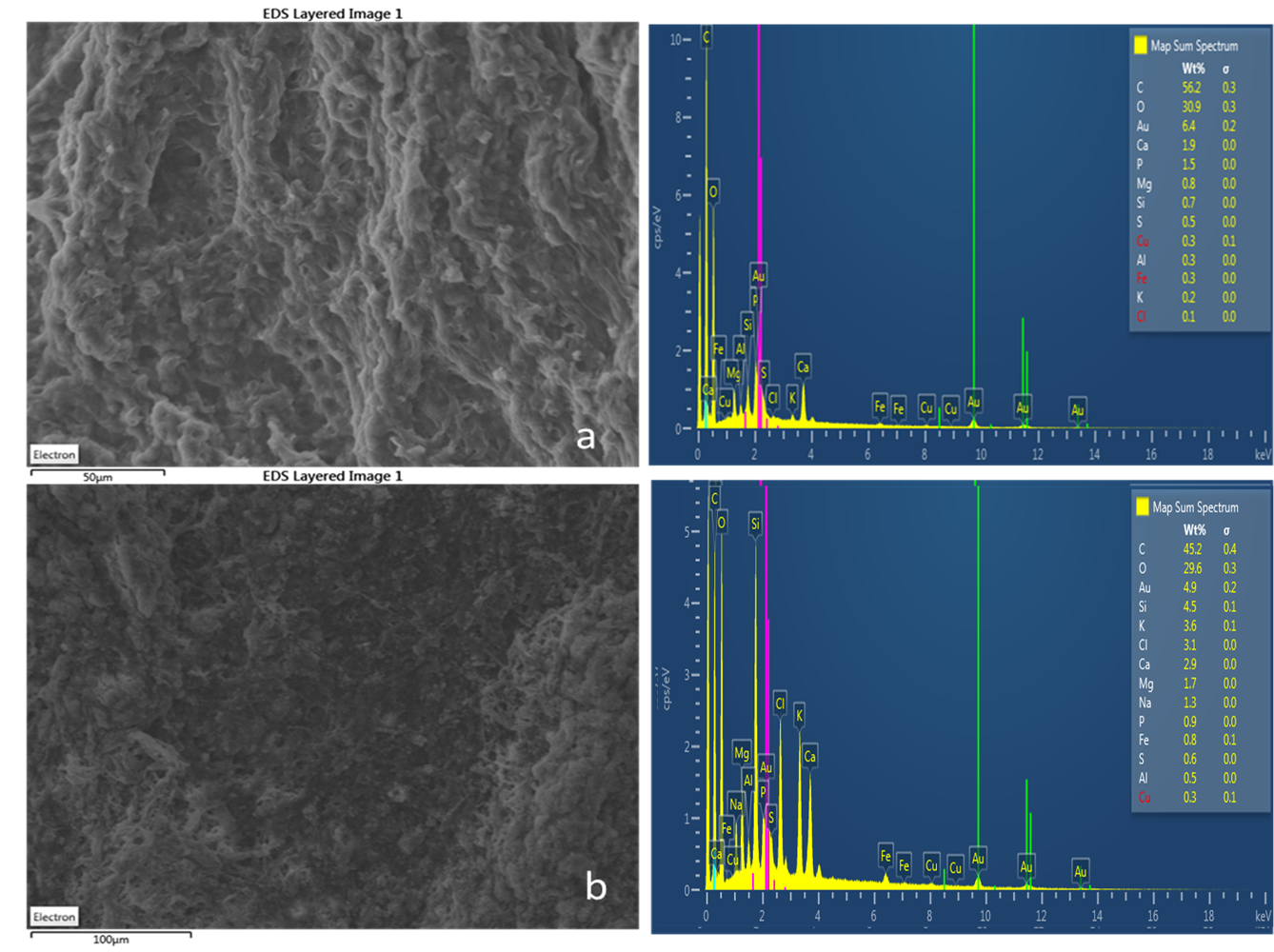


**S. Fig 2a**


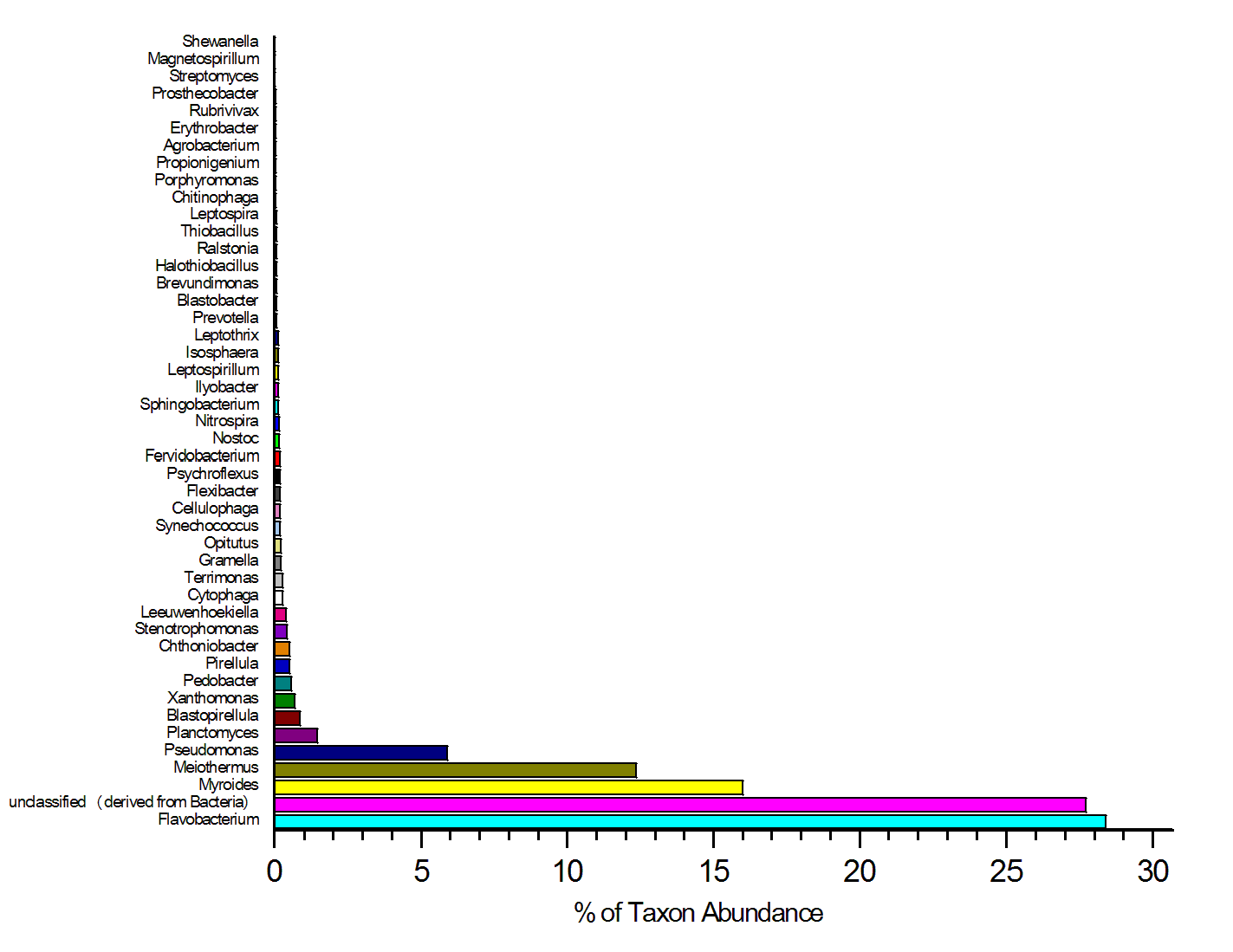


**S. Fig 2b**

**
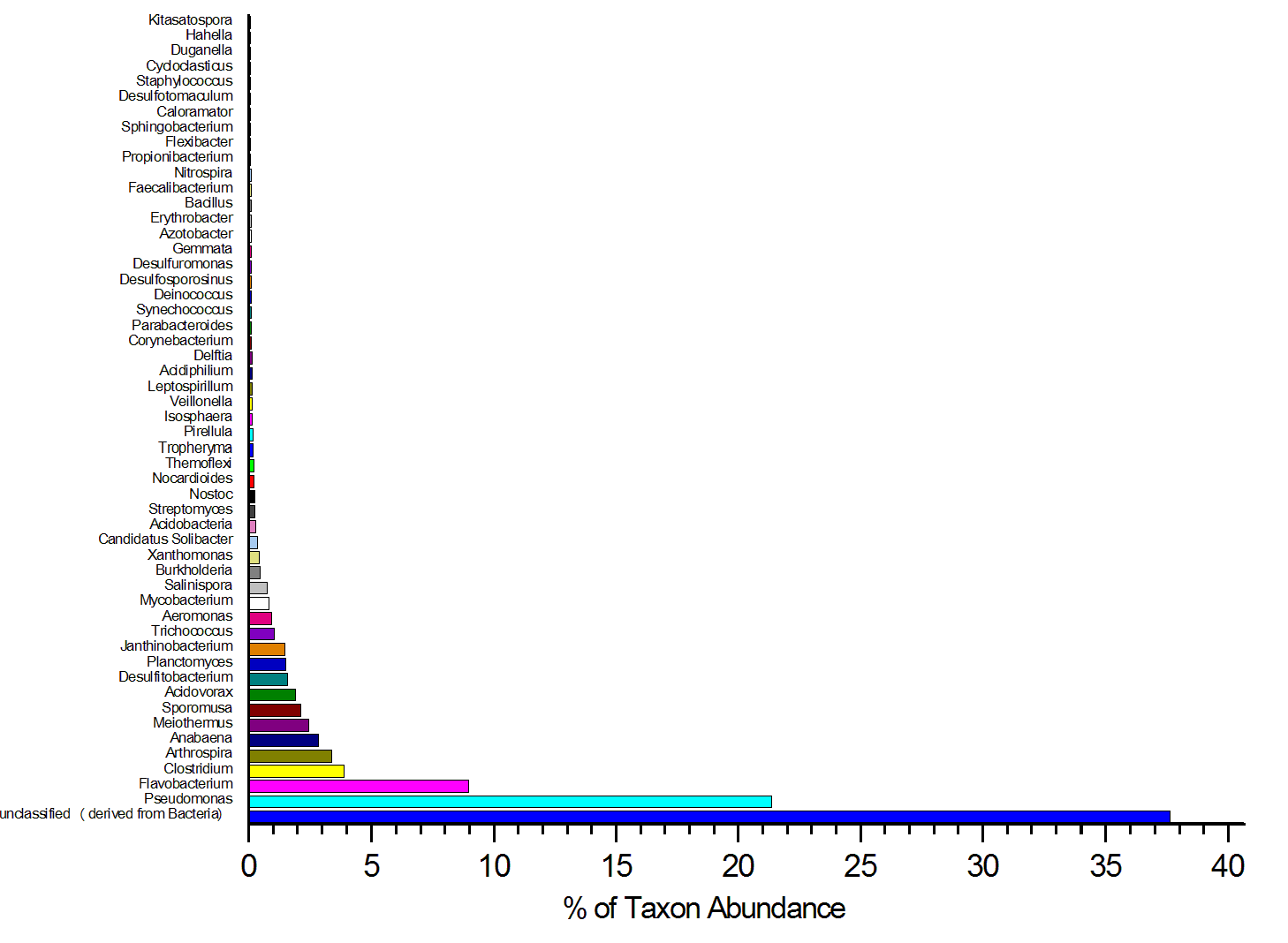
**

**Table S1**

| **Classes (CA)** | **%** | **Classes (BV)** | **%** |
| --- | --- | --- | --- |
| unclassified (derived from Bacteria) | 37.64 | Flavobacteria | 47.3 |
| Gammaproteobacteria | 21.64 | unclassified (derived from Bacteria) | 27.32 |
| Flavobacteria | 8.95 | Deinococci | 12.35 |
| Cyanophyceae | 6.89 | Gammaproteobacteria | 5.9 |
| Betaproteobacteria | 6.68 | Planctomycetacia | 2.97 |
| Clostridia | 3.91 | Betaproteobacteria | 1.35 |
| Solibacteres | 3.3 | Verrucomicrobiae | 0.83 |
| Actinobacteria (class) | 2.65 | Alphaproteobacteria | 0.55 |
| Deinococci | 2.57 | Cytophagia | 0.31 |
| Negativicutes | 1.98 | Nitrospira (class) | 0.31 |
| Planctomycetacia | 1.94 | Thermotogae (class) | 0.21 |
| Alphaproteobacteria | 0.38 | Fusobacteria (class) | 0.21 |
| Epsilonproteobacteria | 0.34 | Deltaproteobacteria | 0.16 |
| Nitrospira (class) | 0.23 | Sphingobacteria | 0.14 |
| Bacteroidia | 0.22 | Spirochaetes (class) | 0.1 |
| Thermoflexia | 0.2 | Actinobacteria (class) | 0.03 |
| Deltaproteobacteria | 0.15 |  |  |
| Cytophagia | 0.13 |  |  |
| Sphingobacteria | 0.11 |  |  |

**Table S2**

| **Calitzdorp mat Genera** | **Phylum** | **% of Sequences** |
| --- | --- | --- |
| unclassified (derived from Bacteria) | Unclassified | 37.64 |
| Pseudomonas | Proteobacteria | 21.35 |
| Flavobacterium | Bacteroidetes | 8.95 |
| Clostridium | Firmicutes | 3.91 |
| Arthrospira | Cyanobacteria | 3.41 |
| Anabaena | Cyanobacteria | 2.85 |
| Meiothermus | Deinococcus–Thermus | 2.45 |
| Sporomusa | Firmicutes | 2.12 |
| Acidovorax | Proteobacteria | 1.93 |
| Desulfitobacterium | Firmicutes | 1.58 |
| Planctomyces | Planctomycetes | 1.5 |
| Janthinobacterium | Proteobacteria | 1.47 |
| Trichococcus | Proteobacteria | 1.04 |
| Aeromonas | Proteobacteria | 0.93 |
| Mycobacterium | Actinobacteria | 0.83 |
| Salinispora | Actinobacteria | 0.77 |
| Burkholderia | Proteobacteria | 0.46 |
| Xanthomonas | Proteobacteria | 0.42 |
| Candidatus Solibacter | Proteobacteria | 0.35 |
| Acidobacteria | Acidobacteria | 0.28 |
| Streptomyces | Actinobacteria | 0.27 |
| Nostoc | Cyanobacteria | 0.27 |
| Nocardioides | Actinobacteria | 0.23 |
| Themoflexi | Chloroflexi | 0.2 |
| Tropheryma | Actinobacteria | 0.17 |
| Pirellula | Planctomycetes | 0.17 |
| Isosphaera | Planctomycetes | 0.15 |
| Veillonella | Firmicutes | 0.13 |
| Leptospirillum | Nitrospirae | 0.13 |
| Acidiphilium | Proteobacteria | 0.13 |
| Delftia | Proteobacteria | 0.13 |
| Corynebacterium | Actinobacteria | 0.12 |
| Parabacteroides | Bacteroidetes | 0.12 |
| Synechococcus | Cyanobacteria | 0.12 |
| Deinococcus | Deinococcus–Thermus | 0.12 |
| Desulfosporosinus | Firmicutes | 0.12 |
| Desulfuromonas | Firmicutes | 0.12 |
| Gemmata | Planctomycetes | 0.12 |
| Azotobacter | Proteobacteria | 0.12 |
| Erythrobacter | Proteobacteria | 0.12 |
| Bacillus | Firmicutes | 0.1 |
| Faecalibacterium | Firmicutes | 0.1 |
| Nitrospira | Nitrospirae | 0.1 |
| Propionibacterium | Actinobacteria | 0.08 |
| Flexibacter | Bacteroidetes | 0.08 |
| Sphingobacterium | Bacteroidetes | 0.08 |
| Caloramator | Firmicutes | 0.08 |
| Desulfotomaculum | Firmicutes | 0.08 |
| Staphylococcus | Firmicutes | 0.08 |
| Cycloclasticus | Proteobacteria | 0.08 |
| Duganella | Proteobacteria | 0.08 |
| Hahella | Proteobacteria | 0.08 |
| Kitasatospora | Actinobacteria | 0.06 |
| Myroides | Bacteroidetes | 0.06 |
| Leptolyngbya | Cyanobacteria | 0.06 |
| Nodularia | Cyanobacteria | 0.06 |
| Oscillochloris | Cyanobacteria | 0.06 |
| Alicyclobacillus | Firmicutes | 0.06 |
| Alkaliphilus | Firmicutes | 0.06 |
| Desulfomicrobium | Firmicutes | 0.06 |
| Lysinibacillus | Firmicutes | 0.06 |
| Paenibacillus | Firmicutes | 0.06 |
| Roseburia | Firmicutes | 0.06 |
| Selenomonas | Firmicutes | 0.06 |
| Acidithiobacillus | Proteobacteria | 0.06 |
| Asticcacaulis | Proteobacteria | 0.06 |
| Erwinia | Proteobacteria | 0.06 |
| Halothiobacillus | Proteobacteria | 0.06 |
| Rhodoplanes | Proteobacteria | 0.06 |
| Rubritepida | Proteobacteria | 0.06 |
| Actinomyces | Actinobacteria | 0.04 |
| Cellulomonas | Actinobacteria | 0.04 |
| Rhodococcus | Actinobacteria | 0.04 |
| Cytophaga | Bacteroidetes | 0.04 |
| Hymenobacter | Bacteroidetes | 0.04 |
| Terrimonas | Bacteroidetes | 0.04 |
| Sphaerospermopsis | Cyanobacteria | 0.04 |
| Acidaminococcus | Firmicutes | 0.04 |
| Dialister | Firmicutes | 0.04 |
| Eubacterium | Firmicutes | 0.04 |
| Teredinibacter | Proteobacteria | 0.04 |
| Synechocystis | Cyanobacteria | 0.02 |
| Lactococcus | Firmicutes | 0.02 |
| Macrococcus | Firmicutes | 0.02 |
| Escherichia | Proteobacteria | 0.02 |
| Magnetospirillum | Proteobacteria | 0.02 |
| Neisseria | Proteobacteria | 0.02 |
| Shewanella | Proteobacteria | 0.02 |
| Symbiobacterium | Proteobacteria | 0.02 |

**Cont…**

| **Brandvlei mat Genera** | **Phylum** | **% of Sequences** |
| --- | --- | --- |
| Flavobacterium | Bacteroidetes | 28.4 |
| unclassified (derived from Bacteria) | Unclassified | 27.72 |
| Myroides | Bacteroidetes | 16.02 |
| Meiothermus | Deinococcus–Thermus | 12.35 |
| Pseudomonas | Proteobacteria | 5.9 |
| Planctomyces | Planctomycetes | 1.45 |
| Blastopirellula | Planctomycetes | 0.86 |
| Xanthomonas | Proteobacteria | 0.72 |
| Pedobacter | Bacteroidetes | 0.6 |
| Pirellula | Planctomycetes | 0.52 |
| Chthoniobacter | Verrucomicrobia | 0.52 |
| Stenotrophomonas | Proteobacteria | 0.45 |
| Leeuwenhoekiella | Bacteroidetes | 0.41 |
| Cytophaga | Bacteroidetes | 0.31 |
| Terrimonas | Bacteroidetes | 0.31 |
| Gramella | Bacteroidetes | 0.24 |
| Opitutus | Verrucomicrobia | 0.24 |
| Synechococcus | Cyanobacteria | 0.22 |
| Cellulophaga | Bacteroidetes | 0.21 |
| Flexibacter | Bacteroidetes | 0.21 |
| Psychroflexus | Bacteroidetes | 0.21 |
| Fervidobacterium | Thermotogae | 0.21 |
| Nostoc | Cyanobacteria | 0.17 |
| Nitrospira | Nitrospirae | 0.17 |
| Sphingobacterium | Bacteroidetes | 0.14 |
| Ilyobacter | Fusobacteria | 0.14 |
| Leptospirillum | Nitrospirae | 0.14 |
| Isosphaera | Planctomycetes | 0.14 |
| Leptothrix | Proteobacteria | 0.14 |
| Prevotella | Bacteroidetes | 0.1 |
| Blastobacter | Proteobacteria | 0.1 |
| Brevundimonas | Proteobacteria | 0.1 |
| Halothiobacillus | Proteobacteria | 0.1 |
| Ralstonia | Proteobacteria | 0.1 |
| Thiobacillus | Proteobacteria | 0.1 |
| Leptospira | Spirocheates | 0.1 |
| Chitinophaga | Bacteroidetes | 0.07 |
| Porphyromonas | Bacteroidetes | 0.07 |
| Propionigenium | Fusobacteria | 0.07 |
| Agrobacterium | Proteobacteria | 0.07 |
| Erythrobacter | Proteobacteria | 0.07 |
| Rubrivivax | Proteobacteria | 0.07 |
| Prosthecobacter | Verrucomicrobia | 0.07 |
| Streptomyces | Actinobacteria | 0.03 |
| Magnetospirillum | Proteobacteria | 0.03 |
| Shewanella | Proteobacteria | 0.03 |
